# Study on miRNAs in Pan-Cancer of the Digestive Tract Based on the Illumina HiSeq System Data Sequencing

**DOI:** 10.1155/2019/8016120

**Published:** 2019-10-15

**Authors:** Chun-hui Lai, Xu-zhi Liang, Xiu-yun Liang, Sheng-jun Ma, Jun-guo Li, Ming-fang Shi, Xu Zhu, Hui-hua Lan, Jiang-hui Zeng

**Affiliations:** ^1^Department of Clinical Laboratory, The Third Affiliated Hospital of Guangxi Medical University/Nanning Second People's Hospital, Nanning, Guangxi Zhuang Autonomous Region, China; ^2^Department of Pathology, The First Affiliated Hospital of Guangxi Medical University, Nanning, Guangxi Zhuang Autonomous Region, China; ^3^Department of Clinical Laboratory, The People's Hospital of Guangxi Zhuang Autonomous Region, Nanning, Guangxi Zhuang Autonomous Region, China

## Abstract

**Objective:**

miRNA has gained attention as a therapeutic target in various malignancies. The proposal of this study was to investigate the biological functions of key miRNAs and target genes in cancers of the digestive tract which include esophageal carcinoma (ESCA), gastric adenocarcinoma (GAC), colon adenocarcinoma (COAD), and rectal adenocarcinoma (READ).

**Materials and Methods:**

After screening differentially expressed miRNAs (DEMIs) and differentially expressed mRNAs (DEMs) in four digestive cancers from The Cancer Genome Atlas (TCGA) database, the diagnostic value of above DEMIs was evaluated by receiver-operating characteristic (ROC) curve analysis. Then, corresponding DEMIs' target genes were predicted by miRWalk 2.0. Intersection of predicted target genes and DEMs was taken as the target genes of DEMIs, and miRNA-mRNA regulatory networks between DEMIs and target genes were constructed. Meanwhile, the univariate Cox risk regression model was used to screen miRNAs with distinct prognostic value, and Kaplan–Meier analysis was used to determine their significance of prognosis. Furthermore, we performed bioinformatics methods including protein-protein interaction (PPI) networks, gene ontology (GO) annotation, Kyoto Encyclopedia of Genes and Genomes (KEGG) pathway enrichment analysis, and gene group RIDA analysis by Gene-Cloud of Biotechnology Information (GCBI) to explore the function and molecular mechanisms of DEMIs and predicted target genes in tumor development.

**Results:**

Eventually, 3 DEMIs (miR-7-3, miR-328, and miR-323a) with significant prognostic value were obtained. In addition, 3 DEMIs (miR-490-3p, miR-133a-3p, and miR-552-3p) and 281 target genes were identified, and the 3 DEMIs showed high diagnostic value in READ and moderate diagnostic value in ESCA, GAC, and COAD. Also, the miRNA-mRNA regulatory network with 3 DEMIs and 281 overlapping genes was successfully established. Functional enrichment analysis showed that 281 overlapping genes were mainly related to regulation of cell proliferation, cell migration, and PI3K-Akt signaling pathway.

**Conclusion:**

The diagnostic value and prognostic value of significant DEMIs in cancers of the digestive tract were identified, which may provide a novel direction for treatment and prognosis improvement of cancers of the digestive tract.

## 1. Introduction

Cancer of the digestive tract is one of the most common malignancies in the world, and it is also a common high-risk malignancy in China, mainly including esophageal carcinoma (ESCA), gastric adenocarcinoma (GAC), colorectal cancer (CRC), and rectal adenocarcinoma (READ). According to the data of the National Cancer Center of China (NCCR) [1], the total number of newly diagnosed cancers of the digestive system in China was 2,089,500 cases and 1,565,500 deaths in 2015. Also, over 2.7 million new cases of cancers of the digestive tract and 1.8 million deaths were estimated to occur in 2018. They were colorectal cancer (6.1%), stomach cancer (5.7%), and esophagus cancer (3.2%) with 1,096,601, 1,033,701, and 572,034 incidences, respectively [2]. The prognosis of cancers of the digestive tract is poor because of the advanced stage at the time of being diagnosed. Thus, early and timely diagnosis is of great importance for patients with cancers of the digestive tract which determines whether the patient can receive radical surgical treatment or not [3, 4]. Therefore, reliable biomarkers are urgently required for detection of the cancers of the digestive tract in primary stages to improve the adverse outcome of patients [5]. In this study, we aim to find potential biomarkers for early diagnosis and explore their molecular mechanisms in cancers of the digestive tract.

MicroRNA (miRNA) is an endogenous noncoding RNA with a length of about 22 nucleotides [6]. It plays an oncogene or tumor suppressor role in biological function by regulating target genes, which can inhibit the translation of protein via binding directly to specific sequences of target gene transcription through regulatory mechanisms including positive regulation of mRNA targets and induction of direct degradation of mRNA [7]. The dysregulated miRNA expression is correlated with human diseases, and some miRNAs also play a causal role in the biological processes of the tumor microenvironment [8]. All the above data indicated that miRNAs have the unique advantages as a biomarker because of their sensitive regulatory function and superior stability. More and more evidence has demonstrated that many key miRNAs participated in the initiation and progression of cancers of the digestive tract [9–11]. Therefore, the purpose of our study was to find the diagnostic and prognostic value of key miRNAs, and the biological function of target genes in cancers of the digestive tract needs to be better elucidated.

In this study, miRNA and mRNA expression profiles associated with ESCA, GAC, COAD, and READ were selected from The Cancer Genome Atlas (TCGA) database. TCGA includes comprehensive cancer genome data such as mutation, copy number variation, mRNA expression, miRNA expression, and methylation data. After identifying differentially expressed miRNAs (DEMIs), we estimated their diagnostic and prognostic value. Furthermore, we take the intersection of differentially expressed genes (DEGs) and the target genes predicted by miRWalk 2.0 and established miRNA-mRNA regulatory networks. Meanwhile, the relationship between miRNAs and their target genes was explored by constructing the regulated network. Furthermore, we investigated the target genes through bioinformatics methods including miRNAs-gene regulatory networks, protein-protein interaction (PPI) networks, gene ontology (GO) annotation, Kyoto Encyclopedia of Genes and Genomes (KEGG) pathway enrichment analysis, and gene group RIDA analysis by Gene-Cloud of Biotechnology Information (GCBI) to explore molecular mechanisms of tumor development and give a new insight into cancers of the digestive tract molecular study.

## 2. Materials and Methods

### 2.1. Collection of DEMIs and DEMs in Cancers

The cancer genome data collected by TCGA can be divided into three levels. Level 1 represents the original sequencing data (fasta, fastq, etc.), level 2 comprises compared bam files, and level 3 comprises processed and standardized data. The Illumina HiSeq system high-throughput miRNA and mRNA expression profile data (level 3) and corresponding survival time clinicopathological data were obtained from TCGA in 2018, which included ESCA, GAC, COAD, and READ tissue samples and corresponding normal tissue samples. Genes with |log_2_ fold change (FC)| > 1 as well as *P* value <0.05 were considered to be DEMIs and differentially expressed mRNAs (DEMs). Since the data are publicly available, additional approval from the ethics committee was not needed in the present study.

### 2.2. Identification of DEMIs and DEMs in the Cancers of the Digestive Tract

The edgeR package in Bioconductor was used to screen the DEMIs and DEMs with |logFC| > 1 and adjusted *P* value < 0.05. We filtered the miRNAs from the above with |logFC| > 3 and adjusted *P* value < 0.05, and then we took the intersection in the upregulated or downregulated miRNAs, respectively, in the cancers of the digestive tract.

### 2.3. Screening of the miRNAs with Prognostic Value

The obtained DEMIs in ESCA, GAC, COAD, and READ were matched with a corresponding survival time and stage. After removing more than 10% of the missing values in above data, DEMIs were analyzed by the univariate Cox risk regression model to screen prognostic factors. Subsequently, we performed intersection of DEMIs associated with the prognosis of ESCA, GAC, COAD, and READ which were identified through the above univariate Cox risk regression model analysis, and DEMIs with significant prognostic value in cancers of the digestive tract were obtained. Kaplan–Meier analysis was used to demonstrate their prognostic significance.

### 2.4. Screening of the miRNAs with Diagnostic Value

DEMIs were extracted (|logFC| > 3, *P* < 0.05) from the above screened differentially expressed miRNAs in ESCA, GAC, COAD, and READ. The diagnostic value of miRNAs was evaluated by ROC curve analysis performed via the MedCalc software.

### 2.5. Prediction of the Target Genes of miRNAs

The potential target genes of miRNAs were predicted by miRWalk 2.0, which is a cross-prediction website that provides experimentally validated miRNA-target genes as well as target genes predicted by binding specific 3′-end-untranslated regions of specific mRNAs and other methods to predict target genes for miRNA. It contains 12 silico databases (miRWalk, Microt4, miRanda, miRBridge, miRDB, miRMap, miRNAMap, PicTar2, PITA, RNA22, RNAhybrid, and TargetScan). The source version and prediction mechanism of these databases are listed in Table 1.

### 2.6. Construction of the miRNA-mRNA Regulatory Networks

Only the genes predicted by 5 or more databases were regarded as potential target genes for each DEMI. The intersection between the predicted genes mentioned above and DEMs of TCGA was considered target genes of DEMIs. Based on the principle of miRNAs negatively regulating their target genes, the miRNA-mRNA regulatory networks were constructed using Cytoscape 3.4.0 which is a public source network visualization as well as visualization analysis tool. The core of the software is the network, and the nodes are genes, proteins, or molecules, and the connection is the interaction between these biological structures.

### 2.7. PPI Network Construction

The STRING database (http://www.string-db.org/) is a large open-source database for searching the direct physical interaction and indirect functional correlation between known proteins and predictive proteins, with 2031 species, 9.6 million protein information, and 13.8 million protein interactions. Its connection lines use different color identifications to distinguish known interactions such as experimentally validated and predicted interactions and others. We constructed the PPI network of the target genes of DEMIs by using it.

### 2.8. GO Annotation, KEGG Pathway Analysis, and RIDA Analysis

To further investigate the functional annotation as well as signal pathways related to the target genes of DEMIs, we conducted GO annotation analysis and KEGG pathway analysis using DAVID (https://david.ncifcrf.gov/), a database with integrated biological information and analytical tools. GO annotation analysis contains 3 categories, including molecular function (MF), biological process (BP), and cellular component (CC), and the hierarchical structure of functional information was described in every catalog. KEGG is second to none in the study of molecular signaling pathways, which includes the GENES database with complete genomic information and sequences as well as the PATHWAY database with more complete functional information. We also performed a gene group RIDA analysis which includes related diseases, core networks, and related transcription factors by applying GCBI (https://www.gcbi.com.cn/). GCBI is a platform which contains more than 1.2 million samples of 20 TB of information data, and it provides many services such as genetic analysis, data analysis, and molecular medical information solution.

### 2.9. Validation by Gene Expression Omnibus DataSets

The clinical roles of the six miRNAs were validated by collecting the relevant miRNA microarrays from Gene Expression Omnibus (GEO) DataSets. Differences in miRNA expression levels between different groups were assessed using Student's *t*-test. The ROC curve analysis of the miRNAs was performed by the MedCalc software.

## 3. Results

### 3.1. DEMIs and DEMs in Cancers of the Digestive Tract on TCGA Database

**Figure 1:**
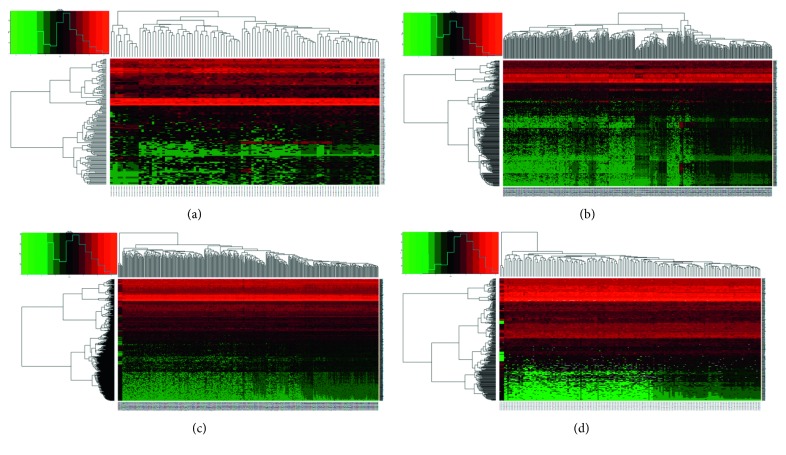
Differential expression of the miRNA heat map in four kinds of cancers of the digestive tract: (a) esophageal carcinoma (ESCA); (b) gastric adenocarcinoma (GAC); (c) colon adenocarcinoma (COAD); (d) rectal adenocarcinoma (READ). Among 157 DEMIs in ESCA tissues, 86 were upregulated and 71 were downregulated, while 252 and 86 were upregulated and downregulated, respectively, in 338 DEMIs in GAC tissues, and 205 were upregulated and 250 were downregulated among the 455 DEMIs in COAD tissues. Of the 339 READ DEMIs, 175 were upregulated and 164 were downregulated.

**Figure 2:**
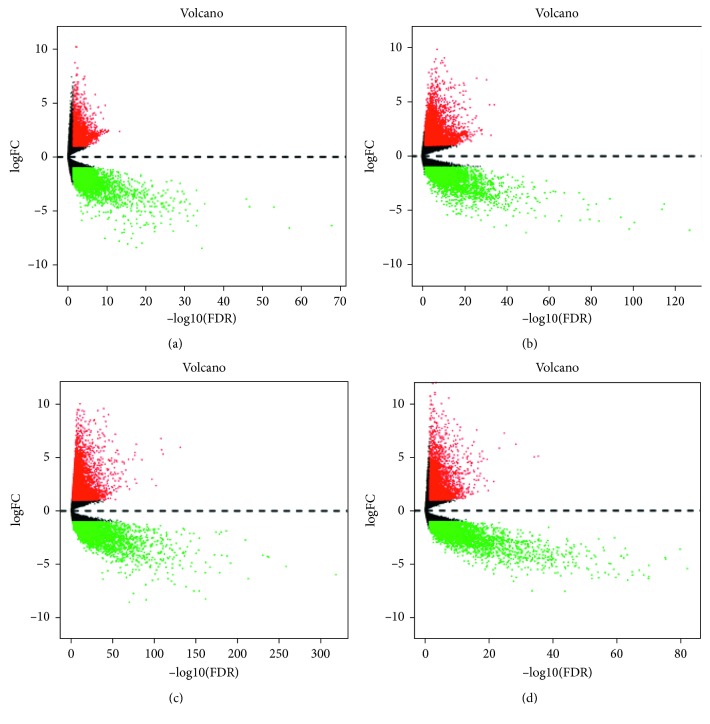
Differential expression of the mRNA volcano map in four kinds of cancers of the digestive tract: (a) esophageal carcinoma (ESCA); (b) gastric adenocarcinoma (GAC); (c) colon adenocarcinoma (COAD); (d) rectal adenocarcinoma (READ). Among 6926 DEMs in ESCA tissues, 2515 were upregulated and 4411 were downregulated, while 11558 and 7922 were upregulated and downregulated, respectively, in 3636 DEMs in GAC tissues, and 205 were upregulated and 250 were downregulated among the 455 DEMs in COAD tissues. Of the 9335 READ DEMs, 5144 were upregulated and 4191 were downregulated. The volcano map was drawn by the R language gplots package. The difference in expression of upregulated miRNAs (logFC > 1, *P* < 0.05) was marked in red, and the difference in expression of downregulated miRNAs (logFC > −1, *P* < 0.05) was marked in green.

After obtaining miRNA expression data of ESCA (90 cases and 13 normal samples), GAC (446 cases and 45 normal samples), COAD (457 cases and 8 normal samples), and READ (162 cases and 3 normal samples), the R language edgeR package was used for difference analysis and screening (|logFC| > 1, *P* < 0.05). The results are shown in Figure 1. In the same way, mRNA expression data of ESCA (80 cases and 11 normal samples), GAC (375 cases and 32 normal samples), COAD (480 cases and 41 normal samples), and READ (167 cases and 10 normal samples) were analyzed. Because of the large amount of data, the DEMs were shown in a more intuitive volcanic map instead of a list (Figure 2).

### 3.2. Prognostic Significance of the DEMIs

**Figure 3:**
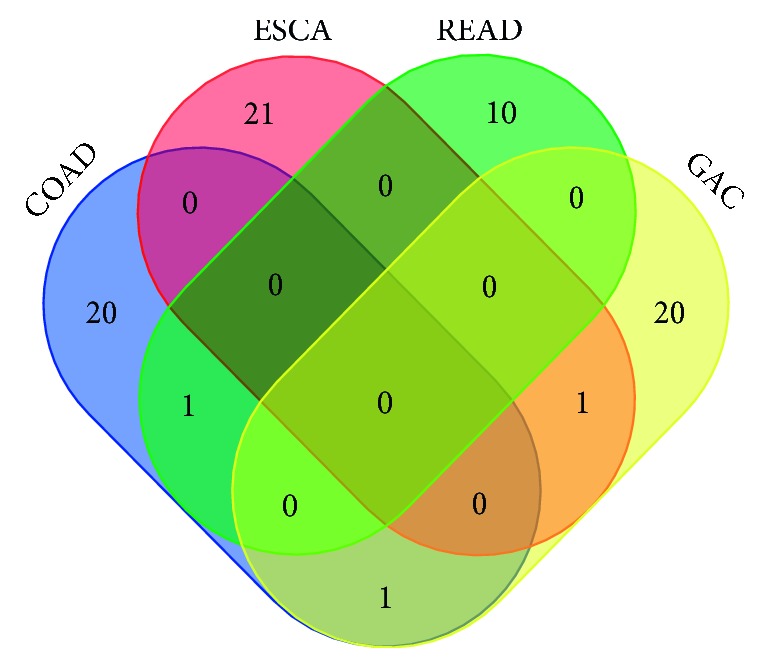
Differential expression of miRNAs associated with the prognosis Venn map in cancers of the digestive tract—colon adenocarcinoma (COAD), esophageal carcinoma (ESCA), rectal adenocarcinoma (READ), and gastric adenocarcinoma (GAC).

We evaluated the prognostic value of three DEMIs by univariate Cox risk regression model analysis. The DEMIs above were further screened by matching with survival time and removing more than 10% missing values. 101 DEMIs related to ESCA, 140 DEMIs related to GAC, 296 DEMIs related to COAD, and 261 DEMIs related to READ were obtained. By identifying a number of prognostic values of miRNA molecular analysis by the univariate Cox risk regression model, DEMIs with prognostic values were obtained, among which 22 were related to ESCA, 22 to GAC, 22 to COAD, and 11 to READ (Table 2). After taking the intersection, miR-7-3 was found in ESCA and GAC intersection, miR-328 in GAC and COAD intersection, and miR-323a in COAD and READ intersection (Figure 3).

**Figure 4:**
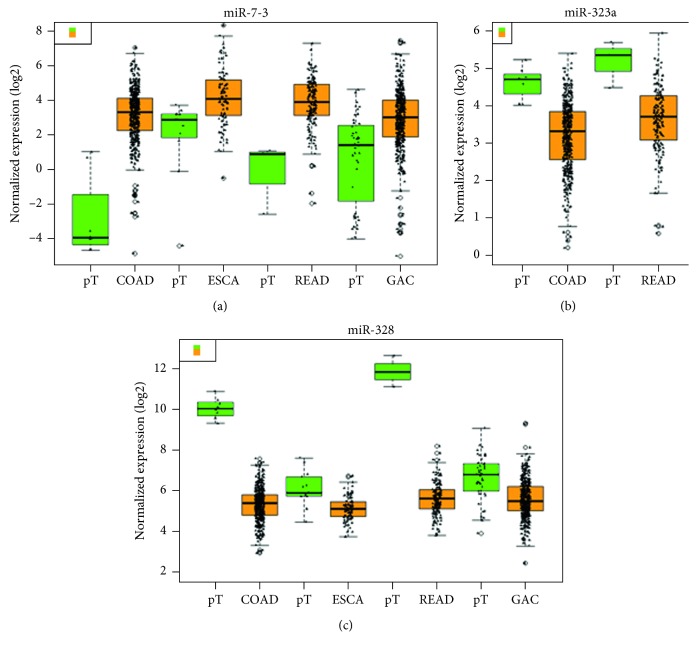
Boxplot of differential expression of (a) miR-7-3, (c) miR-328, and (b) miR-323a in gastrointestinal adenocarcinoma—colon adenocarcinoma (COAD), esophageal carcinoma (ESCA), rectal adenocarcinoma (READ), and gastric adenocarcinoma (GAC). In the boxplot, the abscissa represents the type of cancer and the ordinate represents the log  2 value of the expression. The brownish yellow color represents the tumor sample, and the green color represents the sample of the paracancerous control tissue.

**Figure 5:**
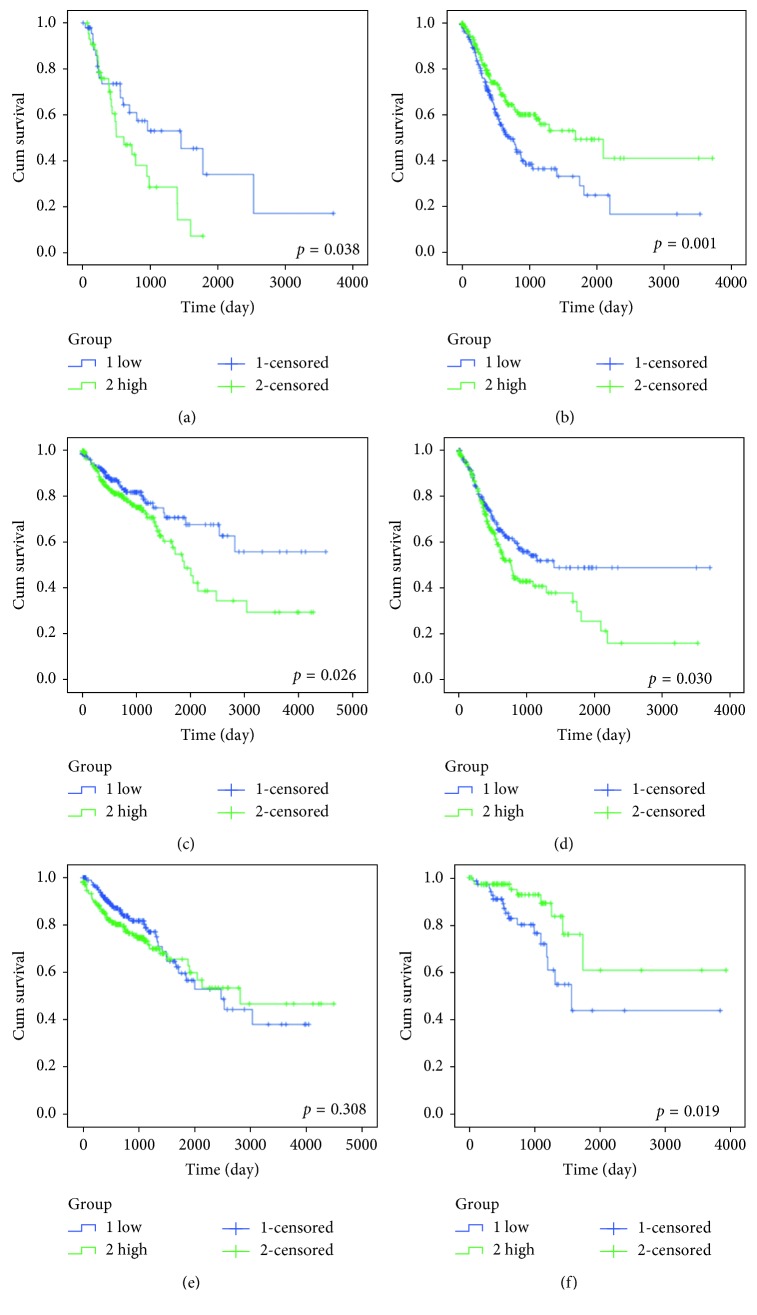
K-M survival curve of miR-7-3, miR-328, and miR-323a in the cancers of the digestive tract—ESCA (esophageal adenocarcinoma), GAC (gastric adenocarcinoma), COAD (colon adenocarcinoma), and READ (rectal adenocarcinoma). K-M survival curve was drawn by the SPSS 19 software. The transverse coordinates represent the survival time (the number of days), and the ordinate represents the cumulative survival function. The blue color represents the low expression of the patient, and the green color represents the high expression of the patient. (a) ESCA miR-7-3. (b) GAC miR-7-3. (c) COAD miR-328. (d) GAC miR-328. (e) COAD miR-323a. (f) READ miR-323a.

We found that the expression level of these 3 miRNAs associated with prognosis in cancers of the digestive tract had significant difference compared with each control sample group. The expression of miR-7-3 was greatly higher than that in four normal sample groups, the expression of miR-328 was all obviously lower than that in four normal sample groups, and the expression of miR-323a was lower in COAD and READ than that in the normal sample group (Figure 4). Subsequently, we performed survival analysis of miR-7-3, miR-328, and miR-323a by Kaplan–Meier analysis, and the results are shown in Figure 5. It was shown that the miR-7-3 high-expression group in ESCA had a lower survival rate than the low-expression group, while the high-expression group of miR-7-3 in GAC had a higher survival rate than the low one, which suggested miR-7-3 was a risk factor in ESCA and a protective factor in GAC. In COAD and GAC, the results indicated that the high-expression group of miR-328 had a lower survival rate than the low one, and miR-328 was a risk factor in COAD and GAC. The K-M survival curve of miR-323a in COAD and READ showed that the miR-323a high-expression group had a higher survival rate than the low one, and miR-323a was a protective factor in READ. The HR value was 1.2207 > 1, suggesting miR-323a as a risk factor in COAD. Meanwhile, the prognostic value of a diversity of clinicopathological parameters was also explored. It was demonstrated by univariate Cox regression analysis that gender, disease stage, N stage, M stage, lymphatic invasion, venous invasion, treatment outcome, neoplasm recurrence, residual tumor, and tumor status were associated with the prognosis of colon cancer (Supplementary [Supplementary-material supplementary-material-1]).

### 3.3. Diagnostic Significance of the DEMIs

**Figure 6:**
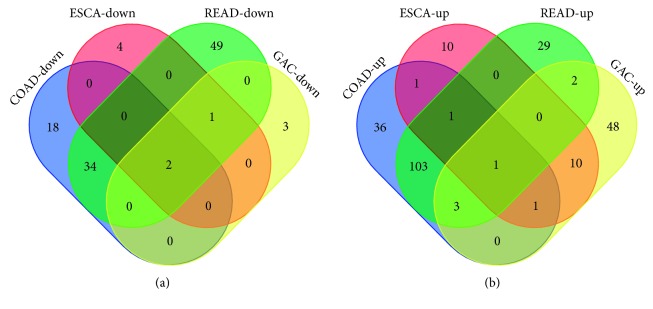
Differential expression of the miRNA (|logFC| > 3, *P* < 0.05) Venn map in adenocarcinoma of the digestive tract—ESCA (esophageal adenocarcinoma), GAC (gastric adenocarcinoma), COAD (colon adenocarcinoma), and READ (rectal adenocarcinoma). The upregulation of miRNAs in 4 kinds of digestive tract tumors obtained 1 intersection (miR-552), and 2 intersections (miR-490 and miR-133a-2) were obtained in downregulated miRNAs.

**Figure 7:**
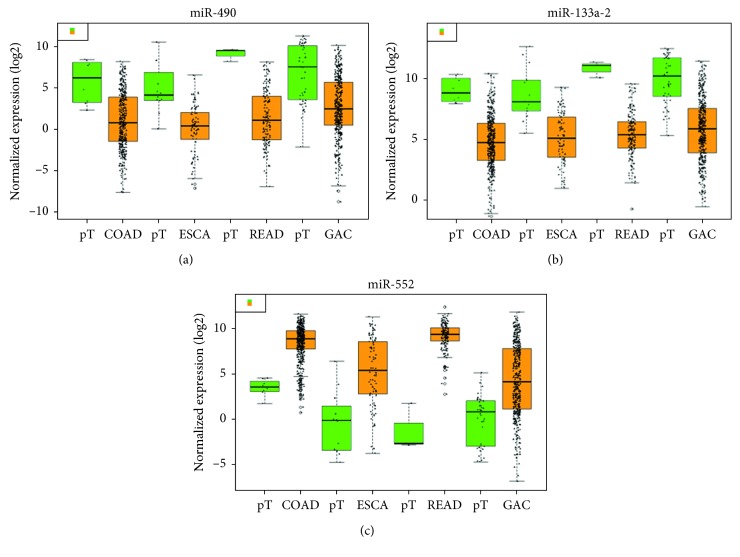
Differential expressions of miR-490, miR-552, and miR-133a-2 in adenocarcinoma of the digestive tract—ESCA (esophageal adenocarcinoma), GAC (gastric adenocarcinoma), COAD (colon adenocarcinoma), and READ (rectal adenocarcinoma). In the boxplot, the abscissa represents the type of cancer and the ordinate represents the log  2 value of the expression. The brownish yellow color represents the tumor sample, and the green color represents the sample of the paracancerous control tissue. (a) miR-490. (b) miR-133a-2. (c) miR-552.

**Figure 8:**
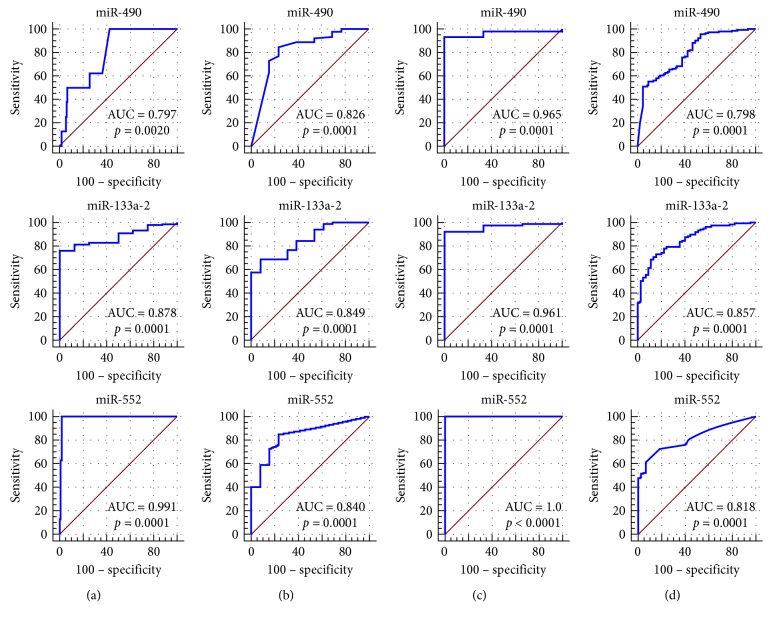
ROC curve of the diagnostic value of miR-490, miR-133a-2, and miR-552 in adenocarcinoma of the digestive tract: (a) COAD (colon adenocarcinoma); (b) ESCA (esophageal carcinoma); (c) READ (rectal adenocarcinoma); (d) GAC (gastric adenocarcinoma). The ROC curve was generated by the MedCalc software. The transversal coordinates represent 100*−*specificity, and the ordinate is sensitivity.

A total of 31 miRNAs (24 upregulated and 7 downregulated) in ESCA, 71 miRNAs (65 upregulated and 6 downregulated) in GAC, 200 miRNAs (146 upregulated and 54 downregulated) in COAD, and 225 miRNAs (139 upregulated and 86 downregulated) in READ were considered DEMIs by the standard of |logFC| > 3 and *P* < 0.05 by the edgeR package. One upregulated miRNA (miR-552) and two downregulated miRNAs (miR-490 and miR-133a-2) were identified as DEMIs according to the intersection taken between upregulated and downregulated miRNAs in the cancers of the digestive tract (Figure 6). We found that the expression level of these 3 miRNAs in cancers of the digestive tract had significant difference compared with each control sample group. The expression of miR-552 was greatly higher than that in the normal sample group, and the expression of miR-490 and miR-133a-2 was obviously lower than that in the normal sample group (Table 3; Figure 7). We evaluated the diagnostic value of the three DEMIs by ROC curve analysis. The AUC of the three miRNAs is shown in Figure 8. The results indicated that three miRNAs exhibited high diagnostic value in READ and moderate diagnostic value in ESCA, GAC, and COAD. Interestingly, the AUC value of the three miRNAs was all biggest in READ, and miR-552 had the highest AUC of 1.0 in READ, which indicated favorable diagnostic value of the three miRNAs in READ.

### 3.4. Prediction of Target Genes and Construction of miRNA-mRNA Regulatory Networks

**Figure 9:**
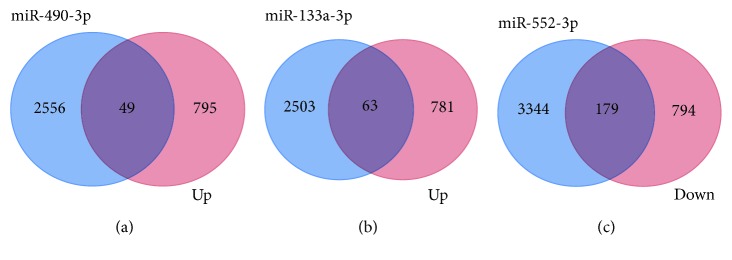
Venn map of predictive target genes of differential miRNAs and common differential genes. 49 target genes were obtained by miR-490-3p, 63 target genes were obtained by miR-133a-3p, and 179 target genes were obtained by miR-552-3p. The light blue color represents the potential target genes for the prediction, the light purple color represents the up- or downregulated common differential genes, and the dark purple color represents the overlapping genes. (a) miR-490-3p. (b) miR-133a-3p. (c) miR-552-3p.

**Figure 10:**
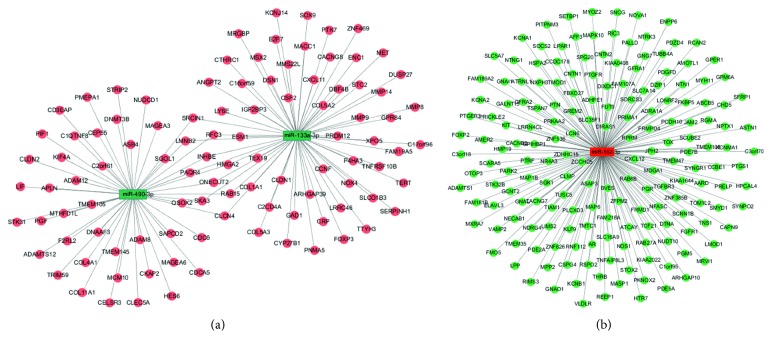
Regulatory networks of miRNA-mRNA: (a) miR-490-3p and miR-133a-3p and (b) miR-552-3p. miRNA-mRNA regulatory networks were visualized by the Cytoscape software. The rectangles and circles represent the miRNAs and target genes, respectively. The green color represents the low expression, and the red color represents the high expression.

**Figure 11:**
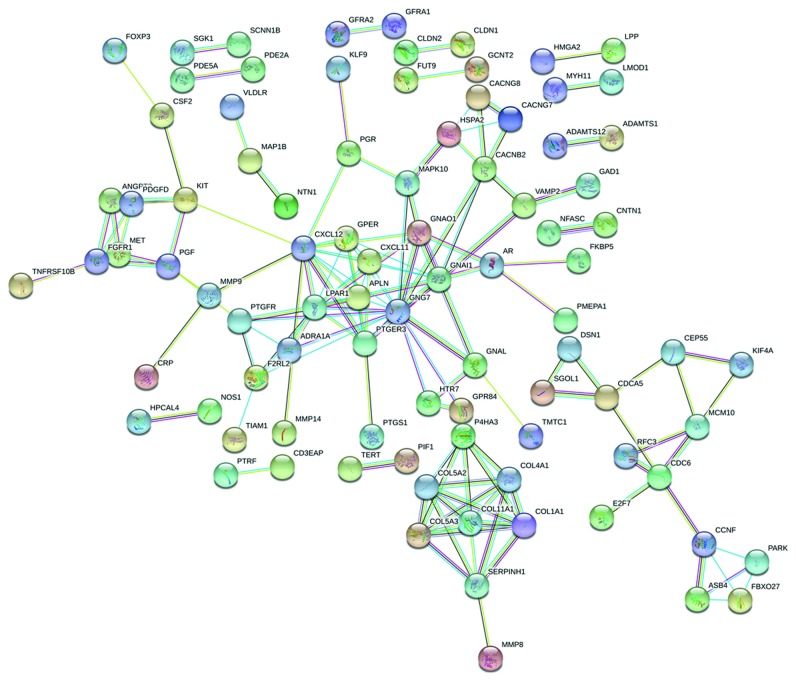
Protein-protein interaction (PPI) network. The interaction between proteins included direct physical interaction and indirect functional correlation. Its connection lines used different color signs to distinguish known interactions, such as experimental and predictive interactions, as well as others. The map has been screened for a single gene interacting with each other.

We predicted potential target genes for miR-490-3p, miR-133a-3p, and miR-552-3p by miRWalk 2.0. Genes that can be predicted in 5 database resources at least were identified as potential target genes to intersect with DEMs obtained from TCGA. Since DEMIs and target genes are negatively correlated under sequence complementation, we intersected the predicted potential target genes of upregulated DEMIs with common downregulated DEMs obtained from TCGA and also the downregulated DEMIs' predicted target genes with common upregulated DEMs. miR-490-3p and miR-133a-3p were intersected with upregulated DEMs. Meanwhile, miR-552-3p and downregulated DEMs were intersected. Finally, 281 overlapped genes in total were obtained (Figure 9). The miRNA-mRNA regulatory networks were established with miR-490-3p and its 49 target genes, miR-133a-3p and its 63 target genes, and miR-552-3p and its 179 target genes using the Cytoscape software (Figure 10). The PPI network was constructed with 281 target genes. According to the frequency of network connection, 22 hub genes (GNG7, CXCL12, GNAI1, LPAR1, PTGER3, APLN, CXCL11, GPER, CACNB2, COL11A1, COL1A1, COL4A1, COL5A2, COL5A3, GNAO1, SERPINH1, CDC6, F2RL2, KIT, P4HA3, PGF, and PTGFR) were obtained (Figure 11).

### 3.5. Biological Function Analysis of Overlapping Genes

**Figure 12:**
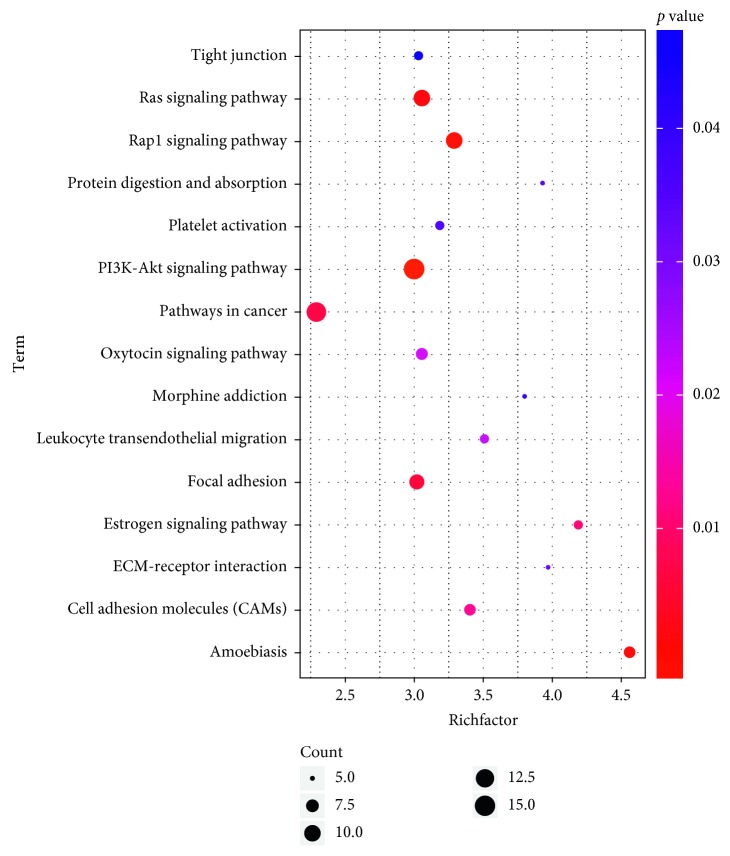
Analysis of the KEGG pathway of the 281 target genes. Top 15 genes had significantly enriched KEGG pathways. All pathways were determined based on the “*Homo sapiens*” category of the KEGG database.

**Figure 13:**
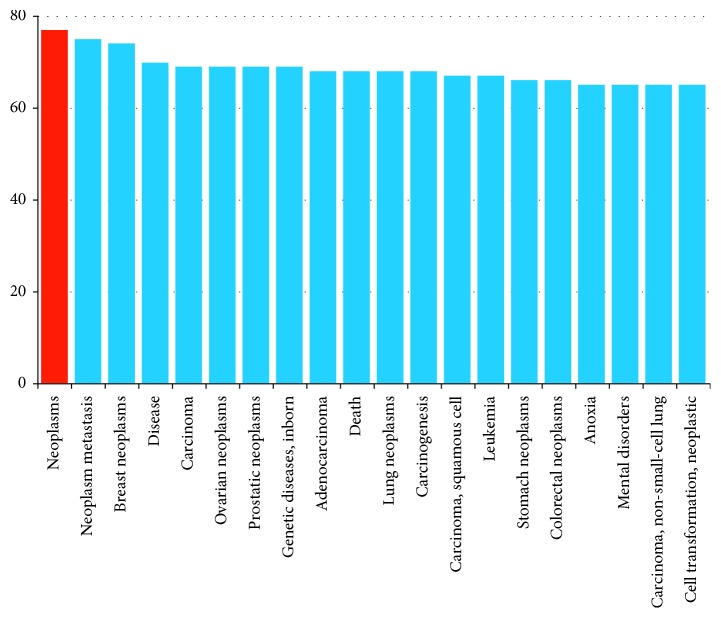
Analysis of related diseases of the 281 target genes. In the analysis of related diseases, it was suggested that these genes are concentrated in tumors, tumor metastasis, adenocarcinoma, death, gastric cancer, colorectal cancer, and other diseases.

**Figure 14:**
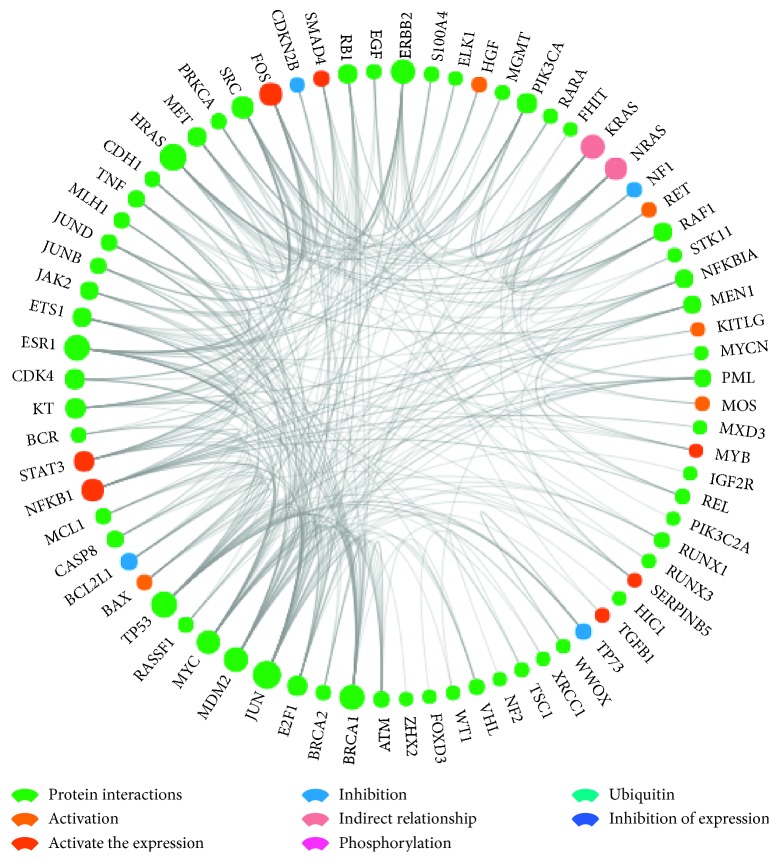
Analysis of the core network. This analysis indicated the interaction, activation, inhibition, activation, expression, inhibition, expression, phosphorylation, ubiquitination, and indirect relationships of genes and gene proteins.

**Figure 15:**
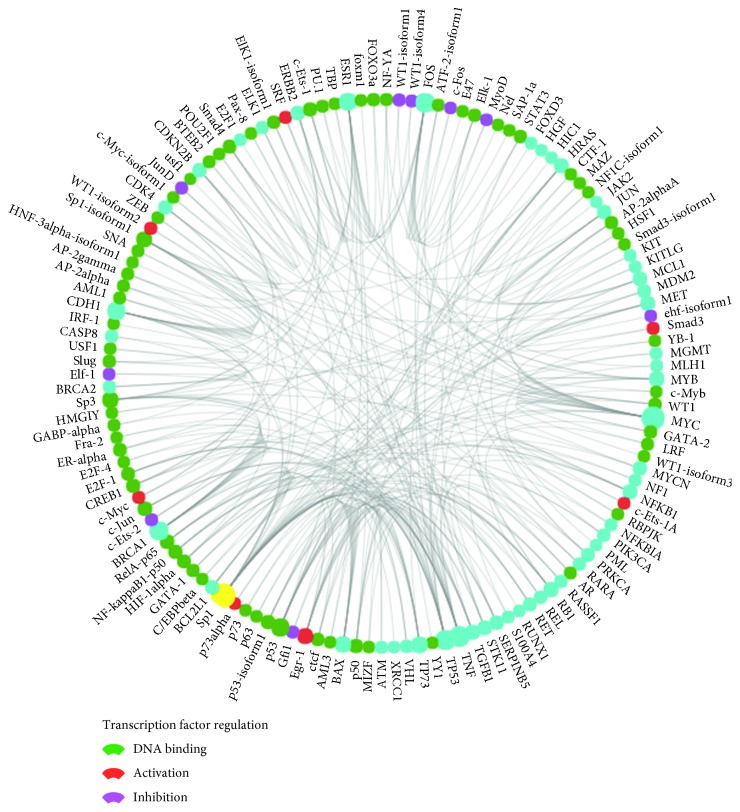
Analysis of related transcription factors. This analysis suggested that the transcription factors regulate DNA binding, transcription factor regulation-activation, and transcription factor regulation-inhibition.

To better understand the biological functions of the 281 overlapping genes, we performed the GO annotation and KEGG pathway analyses. All significant results of GO function analysis with 281 overlapping genes are listed in Table 4. These target genes mainly participate in the biological process (BP) including positive regulation of cell proliferation, cell migration, positive regulation of gene expression, and positive regulation of cell migration. For the term “cellular component” (CC), these genes were involved in the plasma membrane, extracellular region, cell surface, and cell junction. Moreover, the target genes were mainly involved in heparin binding, actin binding, growth factor activity, and extracellular matrix structural constituent classified by molecular function (MF) analysis. Furthermore, 15 significant pathways among these 281 target genes were obtained by KEGG pathway analysis (*P* < 0.05), and the results indicated the target genes were significantly enriched in the PI3K-Akt signaling pathway, Rap1 signaling pathway, Ras signaling pathway, focal adhesion, and pathways in cancer (Table 5; Figure 12). These target genes were also performed for cluster radar analysis, including related diseases, core networks, and related transcription factors in RIDA analysis by GCBI. From the analysis result of related diseases, it was suggested that these genes were associated with tumor metastasis, adenocarcinoma, GAC, and colorectal cancer (CRC) (Figure 13). Core network analysis indicated the relationships of genes and gene proteins with interaction, activation, inhibition, phosphorylation, and ubiquitination (Figure 14). Transcription factor analysis showed that the transcription factor regulation contains DNA binding, activation, and inhibition (Figure 15).

### 3.6. Validation of These miRNAs Using Gene Expression Omnibus DataSets

**Figure 16:**
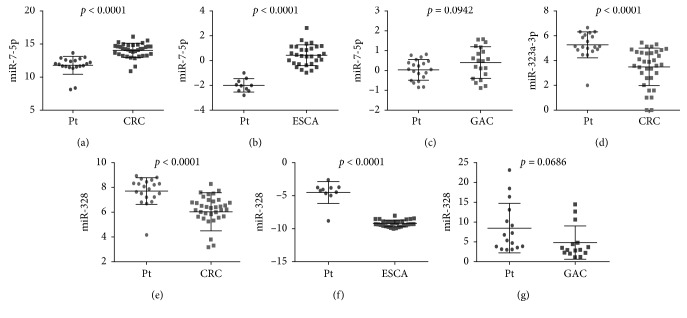
Verification of the differential expression of the three prognostic miRNAs from Gene Expression Omnibus (GEO) DataSets. (a) GSE89974. (b) GSE114110. (c) GSE93415. (d) GSE89974. (e) GSE89974. (f) GSE114110. (g) GSE54397. CRC: colorectal cancer; ESCA: esophageal carcinoma; GAC: gastric adenocarcinoma.

**Figure 17:**
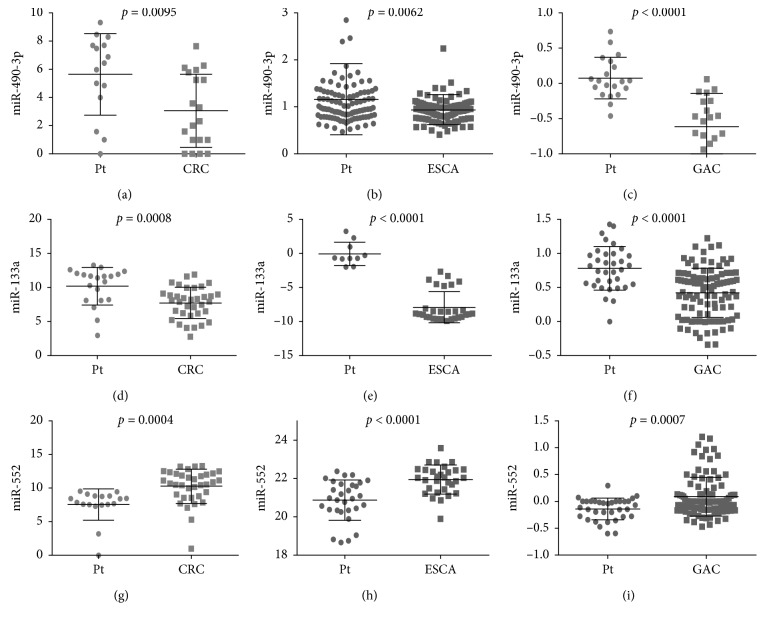
Verification of the expression of the three differentially expressed miRNAs based on Gene Expression Omnibus (GEO) DataSets. (a) GSE89974. (b) GSE55856. (c) GSE93415. (d) GSE89974. (e) GSE114110. (f) GSE30070. (g) GSE89974. (h) GSE66274. (i) GSE30070. CRC: colorectal cancer; ESCA: esophageal carcinoma; GAC: gastric adenocarcinoma.

**Figure 18:**
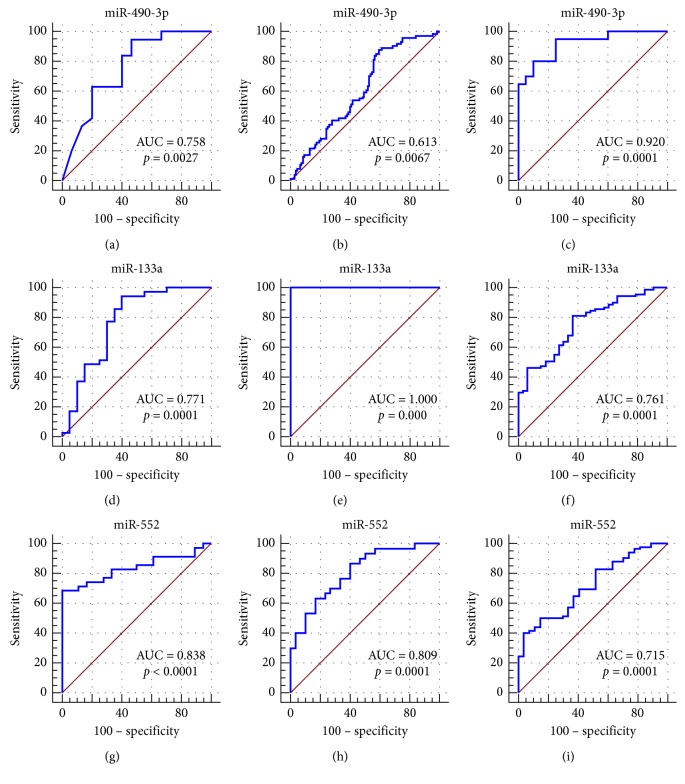
Verification of the diagnostic value of the three differentially expressed miRNAs based on Gene Expression Omnibus (GEO) DataSets. The receiver-operating characteristic (ROC) curves were drawn using the MedCalc software. (a) CRC GSE89974. (b) ESCA GSE55856. (c) GAC GSE93415. (d) CRC GSE89974. (e) ESCA GSE114110. (f) GAC GSE30070. (g) CRC GSE89974. (h) ESCA GSE66274. (i) GAC GSE30070. AUC: area under the ROC curve; CRC: colorectal cancer; ESCA: esophageal carcinoma; GAC: gastric adenocarcinoma.

The expression differences and expression trends of miR-7-5p, miR-323a, and miR-328 in CRC, ESCA, and GAC were verified by GSE89974, GSE114110, GSE93415, and GSE54397. Among them, miR-7-5p was a mature body of miR-7-3 (Figure 16). The expression differences and expression trends of miR-490-3p, miR-133a, and miR-552 in CRC, ESCA, and GAC were verified by GSE89974, GSE55856, GSE93415, GSE114110, GSE30070, and GSE66274 (Figure 17). Furthermore, their diagnostic values were verified by calculating the AUC value. The three areas under the ROC curves (AUCs) of miRNAs (miR-490-3p, miR-133a, and miR-552) were 0.758, 0.613, and 0.920 (CRC, ESCA, and GAC), 0.771, 1.000, and 0.761 (CRC, ESCA, and GAC), and 0.838, 0.809, and 0.715 (CRC, ESCA, and GAC) (Figure 18).

## 4. Discussion

Cancer of the digestive tract is a kind of cancer with high mortality due to its poor prognosis. Increasing evidence has demonstrated that miRNAs played a significant role in diverse tumors and were involved in many biological processes, for instance, proliferation, apoptosis, differentiation, and metabolism [7]. In addition, miRNAs participate in carcinogenesis and progression of malignant tumors by regulation of cell cycle progression, angiogenesis, cell migration, and invasion [12]. A large number of researches have showed that miRNAs play a vital role and could be used as an independent prognosis factor in cancers [13–16]. Some exosomal miRNAs are considered stable biomarkers of tumor disease and play a critical role in carcinogenesis and cancer progression. Exosomal miR-6803-5p could act as a biomarker for diagnosis and prognosis through detecting the level of exosomal miRNA in the serum of the CRC patient [17]. Furthermore, Fu et al. [18] indicated that the circulating miR-17-5p and miR-92a-3p were significantly related to the pathological stage and grade in patients with CRC. Thus, miRNAs have unique advantages to serve as novel biomarkers for cancer diagnosis because of their important regulatory functions, superior stability, and relatively small amounts.

In recent years, more and more studies had been reported from the perspective of pan-cancer [19, 20]. Analysis of scientific problems across multiple cancer types, known as pan-cancer analysis, identifies commonalities and differences in different tumors. Chen et al. presented the characterization of a large number of expressed enhancers in a genome-wide analysis of 8928 tumor samples of 33 cancer types [21]. It was rarely reported from the perspective of pan-cancer to analyze the prognostic and diagnostic value of miRNA in digestive tract tumors. We present the prognostic and diagnostic value of microRNAs in digestive tract tumors from the perspective of pan-cancer.

Our study found that miR-7-3, miR-328, and miR-323a were of significant prognostic value in four cancers of the digestive tract. Recently, Huo found that miR-7-3 was an independent prognostic factor in gastric adenocarcinoma (GAC) by analyzing the clinical data of 393 cases of TCGA and the expression data of 1881 miRNAs via Cox regression and univariate analysis. The results showed that the survival time of the miR-7-3 group with high expression was significantly longer than that of the miR-7-3 group with low one (*P*=0.01) [22], which was consistent with our study. In addition, a study about cervical cancer showed that hsa-miR-7-3 was a potential prognostic biomarker by discovering that increased expression of hsa-miR-7-3 was significantly associated with shortened survival [23], and functional enrichment analysis showed poor prognosis of hsa-miR-7-3-related target genes, which were mainly enriched in the AMPK pathway.

Also, circulating miRNAs can be applied to predict disease progression of primary CRC, including the serum miR-328-3p marker, which was correlated with the overall survival (OS) and disease-free survival (DFS) of phase I–III CRC, and the curative effect of adjuvant chemotherapy was also related with the expression of miR-328-3p [24], which was consistent with our results in COAD. Furthermore, miR-328 overexpression inhibits the invasion of side population (SP) cells in the CRC and reverses drug resistance by targeting matrix metallopeptidase 16 (MMP16) and ATP-binding cassette subfamily G member 2 (ABCG2) [25, 26]. On the contrary, Xue et al. discussed the role of miR-328 as a biomarker to predict tumor recurrence by studying the luciferase activity in the simulant of miR-328 with different concentrations. It was found that miR-328 could target CD44, and they showed a negative regulatory relationship between them, which indicated that detecting the expression level of miR-328 can be used as a means to predict the prognosis of gastric cancer patients [27]. miR-328 has been found to be associated with the prognosis of a variety of tumors in previous studies. In addition to CRC [28], it also involved in non-small-cell lung carcinoma (NSCLC), nasopharyngeal carcinoma, breast cancer, acute myeloid leukemia, glioma, etc. [29–33]. This is similar to the result of Zhang et al. [27], who studied the correlation between five miRNAs (miR-191, miR-28-3p, miR-145, miR-328, and miR-18a) in the serum of NSCLC patients and the three-year overall survival rate of the two groups. The result showed that miR-328 was differentially expressed in paired tumors and normal tissues (*P* < 0.0001), that the target gene of miR-328 was mainly enriched in inflammatory/immune response pathways and pathways related to chemoradiotherapy and/or targeted treatment resistance, and that the miR-328 signal could play a role as a prognostic factor for patients with advanced NSCLC [29, 34]. Also, Ulivi et al. found that PRKCA, il-1, and c-raf1 were involved in the migration of cancer cells through the mRNA target analysis of miR-328 and that the downregulation of hsa-miR-328-3p and its target genes and transcription factors played a key role in the carcinogenesis of NSCLC [35]. Furthermore, in vitro functional tests of Ma cell lines (U251 and U87) showed that high expression of miR-328 could significantly inhibit the proliferation, invasion, and migration of two types of glial cells, and miR-328 could inhibit the invasion and proliferation of malignant cells, indicating a good prognosis for glioma [36]. Similarly, Wu et al. established the expression profile of mRNAs related to miR-328 with 60 GBM samples and identified the gene set closely related to cell mitosis. It was found that the ectopic expression of miR-328 blocked the spreading, adhesion, and proliferation of U87 cells and induced the arrest of the U87 cell division cycle, suggesting the decreased expression of miR-328 in high-level glioma associated with poor survival [33]. In a word, miR-328 can be applied to the diagnosis, prognosis, and treatment of CRC metastasis. Nevertheless, further studies are needed to verify its effectiveness, such as epidemiological and clinical researches.

As for the prognosis factor of miR-323a, there is still no research showing the intrinsic relationship between miR-323a and digestive tract tumors. Our study is the first one to suggest that miR-323a was a protective factor in CRC. But other cancer studies have shown that miR-323a can be used as a prognostic marker for acute coronary syndrome (ACS), cervical cancer, and glioblastoma [37–39]. Pilbrow et al. investigated the level of circulating miRNAs in patients with ACS and studied the relationship between circulating miRNAs and neurohormones, cardiac structure, and function as well as the 5-year follow-up period. It was found that miR-323-3p circulating in ACS patients was significantly increased at least one year later and could be a stable biomarker of ACS [37]. In addition, the increased expression level of miR-323-3p may contribute to the activation of metastatic potential of cervical cancer and may be involved in cell adhesion, cytoskeleton remodeling, and cell proliferation and migration by targeting metastasis-related genes to regulate the steps of the metastasis cascade [38]. In addition, as a prognostic indicator for glioblastoma patients, it has been found that the recurrence of glioblastoma relates to the genes concerning migration and adhesion targeted by miR-323, and the low level of miR-323 is associated with long OS. Furthermore, survival analysis showed that the expression level of miR-323 was increased in patients with nonrecurrence or long recurrence time [39].

Moreover, the diagnostic ability of miRNAs in cancers of the digestive tract has been widely reported. Zeng et al. [40] explored the diagnostic and prognostic value of miRNAs in ESCA based on the TCGA data and found the combined AUC of five miRNAs (miRNA-93, miRNA-21, miRNA-4746, miRNA-196a-1, and miRNA-196a-2) was 0.985. In addition, Wang et al. [41] identified four miRNAs were related to GAC pathogenesis based on 130 patients using qRT-PCR. The AUC values of miR-106a-5p and miR-19b-3p were 0.786 and 0.786, respectively. Similarly, Liu et al. [42] detected the expression of miR-21, miR-29a, miR-92a, miR-125b, and miR-223 in 85 patients with CRC utilizing qRT-PCR, evaluated their diagnostic value, and found the combined AUC of the miRNA was 0.952. Compared with above previous studies, the present study investigated the diagnostic ability of 3 miRNAs (miR-490, miR-133a-2, and miR-552) in pan-cancer of the digestive tract together.

miR-490 has been reported in many digestive tract tumors, such as GAC [43, 44] and CRC [45–47]. Qu et al. [43] detected the expression level of miR-490-3p in GAC patients by qRT-PCR. The result showed that miR-490-3p was downregulated. The expression level of miR-490-3p in *Helicobacter pylori*- (HP-) positive GAC was significantly associated with lymph node metastasis and tumor differentiation. In addition, 5-year overall survival of GAC patients with low miR-490-3p expression was shortened in the HP-positive cohort, and multivariate analysis indicated that low expression of miR-490-3p was an independent risk factor in HP-positive GAC. Also, Tang et al. [48] found that miR-490 expression was downregulated in cancers of the digestive tract by analyzing 1,765 tumor samples in TCGA. The study of Zheng et al. [45] revealed that hypermethylation of the miR-490-3p promoter downregulated the expression of miR-490-3p in colorectal cancer, which revealed that miR-490-3p suppresses the proliferation of tumor cells by inducing apoptosis and inhibits the initiation of epithelial-to-mesenchymal transition (EMT) *in vitro* and *in vivo*, indicating that its expression was continuously downregulated in CRC malignancy and CRC cell lines. The ability of migration and invasion in tumor cells was inhibited if miR-490-3p was oversuppressed, and the opposite effect was produced by knocking down miR-490-3p. Further studies found that it can inhibit CRC cell migration and invasion by targeting the TGF-β signaling pathway [46]. For miR-133a-2, however, the study of its function in tumors is rare in recent years, and only GAC [49], CRC [50], and bladder cancer [51] have been reported. Chen et al. [49] found that the AUC of miR-133a-2 in GAC was 0.905, and the sensitivity and specificity were 76.75% and 92.68% based on the TCGA data. miR-133a-2 showed significant correlation between race, tumor grade, tumor pathology, metastasis, and tumor stage (*P* < 0.05). In addition, miR-133a-2 was less expressed in the early stage (T1/T2 grade and I/II tumor stage) and was significantly higher in the late stage (*P* < 0.05), which suggested that miR-133a-2 may be a new potential predictor in the tumor stage. miR-552 function in CRC has been widely reported [52–56], and it has also been studied in lung tumor [57], breast tumor [58], and pancreatic cancer [59]. Wang and Liu [52] suggested that miR-552 was highly expressed in CRC through qRT-PCR experiments on 183 CRC patients and precancerous tissues, and high expression of miR-552 was significantly related to histological grade, lymph node metastasis, and TNM stage. Overall survival in patients with high expression levels of miR-552 was obviously lower than that in patients with low expression levels of miR-552 (*p*=0.0036), which indicated high expression of miR-552 was an independent prognostic factor for CRC patients with poor prognosis through univariate and multivariate analysis. Cao et al. [53] found that miR-552 was upregulated in CRC tissues and cell lines by qRT-PCR experiments in 20 pairs of CRC patient tissues and adjacent control tissues and cell lines. miR-552 could be applied as a potential predictor and diagnostic biomarker for CRC. Wang et al. [54] also found that miR-552 was highly expressed in CRC by similar experiments and found that miR-552 promotes CRC metastasis by targeting ADAM28, which suggested miR-552 may serve as a new target for CRC therapy. miR-552 was highly expressed in CRC tissues in analysis of 25 cases of CRC and 10 cases of normal colorectal using qRT-PCR [56], and it was found to be related to the clinical stage and distant metastasis (*p* < 0.05).

In this study, 3 DEMIs (miR-490, miR-133a-2, and miR-552) exhibited high diagnostic ability in READ and moderate diagnostic ability in ESCA, GAC, and COAD. The expressions of miR-490 and miR-133a-2 were downregulated, and miR-552 was upregulated in cancers of the digestive tract. Our results were consistent with the former studies and validated through analysis of the Gene Expression Omnibus (GEO) DataSets. To summarize, the significant prognostic value of miR-490, miR-133a-2, and miR-552 in ESCA, GAC, COAD, and READ provided solid reasons for being therapeutic targets for patients with pan-cancer of the digestive tract, for which the novel prognosis and candidates may serve certain functions in process of tumor development, as predicted.

Increasing evidence has demonstrated that new diagnostic or therapeutic targets could be obtained, and new insight into the molecular mechanisms of tumor development may be provided through network regulation and signaling pathway analysis [40, 45, 53, 60, 61]. So we construct the miRNA-gene network and further explore the biological process and pathways in which the target genes could be participated. We perform the PPI network analysis, GO annotation and KEGG pathway analysis, and gene group RIDA analysis. 22 hub genes were identified by PPI network analysis, and G protein *γ* subunit 7 (GNG7) was most active. GNG7 is the target gene of miR-552, but the relationship between GNG7 and miR-552 is unclear. The team of surgeons at Kyushu University in Japan has explored the role of GNG7 in different tumors. They found that GNG7 was downregulated in pancreatic cancer, gastric cancer, intrahepatic cholangiocarcinoma, esophageal cancer, and CRC [62–65]. They also found that the expression of GNG7 was significantly repressed by miR-328 and the suppression of GNG7 was associated with great invasiveness and poor prognosis [65]. Then, we supposed that the upregulated miR-552 might suppress the expression of GNG7 to promote the development of cancers of the digestive tract; however, this hypothesis needs to be confirmed with in vitro and in vivo experiments in future. In these hub genes, CXCL12 has been reported to be related to poor prognosis in ESCA [66]. We previously identified CXCL12 as one of the hub genes in ESCA with 187 cases based on TCGA data [40]. The GO annotation analysis of the 281 overlapping genes suggested that the target genes mainly participate in positive regulation of cell proliferation and play their molecular role by heparin binding. The KEGG pathways were also identified, such as PI3K-Akt signaling pathway, Rap1 signaling pathway, and pathways in cancer, which play a key role in the occurrence and development of cancers of the digestive tract. In the gene group RIDA analysis, the target genes were related to neoplasm, neoplasm metastasis, stomach neoplasm, and colorectal neoplasm. In addition, analysis of the core network indicated the relationship of activation, inhibition, and ubiquitination between these target genes. In the analysis of related transcription factors, the target genes were significantly enriched in DNA binding, activation, and inhibition. All the results of bioinformatics analysis demonstrated that these three miRNAs might influence the tumorigenesis and deterioration of tumors by targeting manifold genes and pathways. The target genes might play their role in the occurrence and development of cancers of the digestive tract through a number of biological processes, gene and gene interconnections, and the regulation of transcription factors. The results in this present study gave us a direction to further explore the molecular mechanisms of the three miRNAs in the development of cancers of the digestive tract by using the experimental technique.

## 5. Conclusion

To summarize, we identified three DEMIs (miR-7-3, miR-328, and miR-323a) which are of significant prognostic value and three DEMIs (miR-490-3p, miR-133a-3p, and miR-552-3p) with high diagnostic ability in READ and moderate diagnostic ability in ESCA, GAC, and COAD by analyzing the miRNA expression profiles in a large number of samples from the TCGA database and GEO DataSet. A total of 281 target genes were also found, and they were used to perform a number of biological function analyses. The results indicated that variant genes and signaling pathways were targeted by these three DEMIs in cancers of the digestive tract. However, *in vitro* and *in vivo* experimentally functional verification was needed for target gene functional analysis in the present study. From the perspective of pan-cancer, our study may provide significant contribution to validation studies and bring the diagnostic and prognostic miRNAs or target genes to clinics in the future.

## Figures and Tables

**Table 1:** miRWalk 2.0's 12 database lists.

Resource	Version	Information
Putative miRNA-target interaction information		
DIANA-microT (miRWalk)	4.0 and 5.0	miRNA binding sites within 3′-UTR
DIANA-microT-CDS (Microt4)	5.0	miRNA binding sites within CDS
miRanda	August 2010	Locally executed for promoter, CDS, 5′- and 3′-UTR, and mitochondrial genomes and miRNA-miRNA interactions
miRBridge	4.0	miRNA binding sites within 3′-UTR
miRDB	4.0	miRNA binding sites within 3′-UTR
miRMap	January 2013	miRNA binding sites within 3′-UTR
miRNAMap	September 2008	miRNA binding sites within 3′-UTR
doRiNA (PicTar2)	2.0	miRNA binding sites within 3′-UTR
PITA	November 2007	miRNA binding sites in CDS and 5′- and 3′-UTR, while locally executed for mitochondrial genes and miRNA-miRNA interactions
RNA22	2.0	miRNA binding sites within CDS and 5′- and 3′-UTR
RNAhybrid	2.1	Locally executed for promoter, CDS, 5′- and 3′-UTR, and mitochondrial genomes and miRNA-miRNA interactions
TargetScan	6.1	Locally executed for promoter, CDS, 5′- and 3′-UTR, and mitochondrial genomes and miRNA-miRNA interactions

**Table 2:** Differential expression of miRNAs associated with the prognosis of ESCA, GAC, COAD, and READ.

Cancer	Name	HR	Lower	Higher	*P*
ESCA	miR-23b	0.589	0.422	0.821	0.002
	miR-550a-1	1.6032	1.193	2.154	0.002
	miR-29c	0.7028	0.559	0.883	0.003
	miR-550a-3	1.6423	1.18	2.285	0.003
	miR-550a-2	1.5782	1.13	2.205	0.007
	miR-3682	1.5101	1.114	2.047	0.008
	miR-3074	1.5073	1.106	2.054	0.009
	miR-421	1.5859	1.113	2.259	0.011
	miR-503	1.432	1.084	1.892	0.012
	miR-1301	1.5588	1.1	2.21	0.013
	miR-7-2	1.2644	1.045	1.53	0.016
	miR-7-3	1.2542	1.039	1.514	0.018
	miR-18a	1.3229	1.043	1.678	0.021
	miR-483	1.1883	1.025	1.377	0.022
	miR-30e	0.5741	0.353	0.934	0.025
	miR-935	1.1622	1.017	1.329	0.028
	miR-767	1.066	1.005	1.131	0.033
	miR-378c	0.7493	0.575	0.976	0.033
	miR-28	0.6329	0.414	0.968	0.035
	miR-143	0.7696	0.603	0.983	0.036
	miR-133b	0.9134	0.839	0.995	0.038
	miR-301b	1.2931	1.004	1.665	0.046

GAC	miR-328	1.2993	1.111	1.519	0.001
	miR-7-3	0.8867	0.824	0.954	0.001
	miR-125a	1.3635	1.125	1.652	0.002
	miR-365a	1.3167	1.082	1.603	0.006
	miR-96	0.8544	0.764	0.956	0.006
	miR-675	1.0864	1.024	1.152	0.006
	miR-365b	1.3137	1.079	1.599	0.007
	miR-877	0.909	0.847	0.976	0.008
	miR-708	1.1873	1.043	1.351	0.009
	miR-100	1.1213	1.027	1.224	0.01
	miR-200b	0.8592	0.764	0.966	0.011
	miR-200c	0.8546	0.754	0.969	0.014
	miR-145	1.126	1.022	1.241	0.017
	miR-548v	1.1094	1.017	1.21	0.019
	miR-217	1.1241	1.019	1.241	0.02
	miR-30a	1.153	1.021	1.302	0.022
	miR-218-2	1.131	1.014	1.262	0.027
	miR-218-1	1.1291	1.013	1.259	0.029
	miR-187	1.0563	1.006	1.109	0.029
	miR-139	1.1427	1.009	1.294	0.036
	miR-216a	1.0778	1.005	1.156	0.036
	miR-125b-1	1.111	1.001	1.233	0.049

COAD	miR-6854	0.8514	0.778	0.932	0
	miR-3677	0.7493	0.633	0.887	0.001
	miR-887	1.3203	1.099	1.586	0.003
	miR-149	1.2631	1.065	1.497	0.007
	miR-126	1.5382	1.122	2.11	0.008
	miR-144	0.7959	0.673	0.942	0.008
	miR-130a	1.3814	1.082	1.764	0.01
	miR-200a	0.7478	0.596	0.938	0.012
	miR-3917	0.8773	0.789	0.975	0.015
	miR-486-2	0.8259	0.701	0.972	0.022
	miR-32	1.4018	1.05	1.872	0.022
	miR-34b	1.1443	1.018	1.286	0.024
	miR-328	1.3304	1.035	1.709	0.026
	miR-1229	1.1214	1.014	1.24	0.026
	miR-495	1.3473	1.034	1.756	0.027
	miR-337	1.3454	1.027	1.762	0.031
	miR-4999	1.1411	1.01	1.289	0.033
	miR-549a	0.8992	0.815	0.992	0.034
	miR-136	1.24	1.01	1.523	0.04
	miR-26b	1.3793	1.004	1.894	0.047
	miR-4746	0.902	0.815	0.998	0.047
	miR-323a	1.2207	1.001	1.488	0.048

READ	miR-942	0.5285	0.341	0.819	0.004
	miR-190a	0.6133	0.432	0.871	0.006
	miR-664b	2.3182	1.262	4.259	0.007
	miR-1226	1.5067	1.055	2.152	0.024
	miR-29b-1	1.5517	1.054	2.285	0.026
	miR-889	0.5699	0.347	0.937	0.027
	hsa-let-7d	3.3399	1.109	10.06	0.032
	miR-323a	0.6935	0.496	0.97	0.033
	miR-381	0.6315	0.409	0.975	0.038
	miR-98	1.8075	1.028	3.178	0.04
	miR-382	0.5242	0.28	0.98	0.043

**Table 3:** Differential expression of miR-552, miR-490, and miR-133a-2 in adenocarcinoma of the digestive tract.

ID	Cancer	logFC	logCPM	*P* value	FDR
miR-552	ESCA	4.917785	5.680932	3.79*E*−05	0.000781397
	GAC	6.029278	5.575829	4.84*E*−18	1.81*E*−16
	COAD	5.570729	7.551953	1.39*E*−11	1.46*E*−10
	READ	9.169904	7.788333	7.80*E*−11	1.43*E*−09

miR-490	ESCA	−5.21591	2.65515	5.31*E*−22	2.84*E*−19
	GAC	−3.36686	4.719789	2.47*E*−25	1.69*E*−23
	COAD	−3.30811	2.495235	9.38*E*−07	5.95*E*−06
	READ	−5.02312	3.0164	1.22*E*−09	1.88*E*−08

miR-133a-2	ESCA	−3.91342	5.746671	1.07*E*−21	3.87*E*−19
	GAC	−3.19544	6.478735	3.01*E*−39	7.88*E*−37
	COAD	−3.09357	4.728642	2.56*E*−12	2.86*E*−11
	READ	−4.4379	5.079706	2.22*E*−17	1.09*E*−15

**Table 4:** GO function analysis of the 281 target genes (*P* < 0.05).

Category	Term	Count	*P* value	Fold enrichment
GOTERM_BP	GO:0030574∼collagen catabolic process	8	4.16*E* − 05	8.463709677
GOTERM_BP	GO:0001764∼neuron migration	9	1.60*E* − 04	5.803686636
GOTERM_BP	GO:0008284∼positive regulation of cell proliferation	19	1.94*E* − 04	2.760695002
GOTERM_BP	GO:0035987∼endodermal cell differentiation	5	6.14*E* − 04	12.53882915
GOTERM_BP	GO:0048863∼stem cell differentiation	5	6.14*E* − 04	12.53882915
GOTERM_BP	GO:0071560∼cellular response to transforming growth factor beta stimulus	6	7.44*E* − 04	8.290980908
GOTERM_BP	GO:0016477∼cell migration	10	0.0010267	3.936609152
GOTERM_BP	GO:0043410∼positive regulation of MAPK cascade	7	0.0012322	5.851453604
GOTERM_BP	GO:0043406∼positive regulation of MAP kinase activity	6	0.0017338	6.885729907
GOTERM_BP	GO:0010628∼positive regulation of gene expression	12	0.0017773	3.101206599
GOTERM_BP	GO:0010976∼positive regulation of neuron projection development	7	0.002002	5.325480246
GOTERM_BP	GO:0030199∼collagen fibril organization	5	0.0025085	8.680727874
GOTERM_BP	GO:0010564∼regulation of cell cycle process	3	0.0031088	33.85483871
GOTERM_BP	GO:0007165∼signal transduction	30	0.003613	1.749604068
GOTERM_BP	GO:0001666∼response to hypoxia	9	0.0040009	3.542948237
GOTERM_BP	GO:0031100∼organ regeneration	5	0.004964	7.203157172
GOTERM_BP	GO:0010954∼positive regulation of protein processing	3	0.0056913	25.39112903
GOTERM_BP	GO:0060346∼bone trabecula formation	3	0.0056913	25.39112903
GOTERM_BP	GO:0002040∼sprouting angiogenesis	4	0.0056932	10.83354839
GOTERM_BP	GO:0030335∼positive regulation of cell migration	9	0.0059884	3.311886396
GOTERM_BP	GO:0050680∼negative regulation of epithelial cell proliferation	5	0.0092262	6.045506912
GOTERM_CC	GO:0005581∼collagen trimer	8	3.46*E* − 04	6.071630851
GOTERM_CC	GO:0045202∼synapse	10	0.0011938	3.85766601
GOTERM_CC	GO:0008076∼voltage-gated potassium channel complex	7	0.0016247	5.554162313
GOTERM_CC	GO:0005887∼integral component of plasma membrane	35	0.0019126	1.727089341
GOTERM_CC	GO:0005576∼extracellular region	38	0.0025978	1.648014088
GOTERM_CC	GO:0005886∼plasma membrane	79	0.0033028	1.338528665
GOTERM_CC	GO:0005925∼focal adhesion	14	0.0041987	2.500083292
GOTERM_CC	GO:0009986∼cell surface	17	0.0050176	2.190043969
GOTERM_CC	GO:0005578∼proteinaceous extracellular matrix	11	0.0052624	2.865900383
GOTERM_CC	GO:0031225∼anchored component of membrane	7	0.0056457	4.325365341
GOTERM_CC	GO:0030054∼cell junction	15	0.0063833	2.281822052
GOTERM_CC	GO:0030018∼Z disc	7	0.0069446	4.142087149
GOTERM_CC	GO:0044224∼juxtaparanode region of axon	3	0.0084607	20.94712644
GOTERM_CC	GO:0043197∼dendritic spine	6	0.0142907	4.189425287
GOTERM_CC	GO:0030424∼axon	9	0.014609	2.830692762
GOTERM_CC	GO:0043025∼neuronal cell body	11	0.0153854	2.43828985
GOTERM_CC	GO:0005788∼endoplasmic reticulum lumen	8	0.0205088	2.909323116
GOTERM_CC	GO:0030425∼dendrite	11	0.0225323	2.292720307
GOTERM_CC	GO:0005923∼bicellular tight junction	6	0.0229941	3.707456007
GOTERM_CC	GO:0016324∼apical plasma membrane	10	0.0242207	2.399441745
GOTERM_CC	GO:0042383∼sarcolemma	5	0.0333821	4.107279693
GOTERM_CC	GO:0005588∼collagen type V trimer	2	0.0421953	46.54916986
GOTERM_MF	GO:0008201∼heparin binding	11	9.92*E* − 05	4.815637967
GOTERM_MF	GO:0005201∼extracellular matrix structural constituent	6	0.0026279	6.272744163
GOTERM_MF	GO:0004222∼metalloendopeptidase activity	7	0.0055384	4.339110638
GOTERM_MF	GO:0008083∼growth factor activity	8	0.0085235	3.459044106
GOTERM_MF	GO:0001948∼glycoprotein binding	5	0.013712	5.388126396
GOTERM_MF	GO:0003779∼actin binding	10	0.0182082	2.519627452
GOTERM_MF	GO:0031683∼G-protein beta/gamma-subunit complex binding	3	0.0323213	10.50684647
GOTERM_MF	GO:0004114∼3′,5′-cyclic-nucleotide phosphodiesterase activity	3	0.0418623	9.136388237
GOTERM_MF	GO:0016167∼glial cell-derived neurotrophic factor receptor activity	2	0.0420504	46.69709544
GOTERM_MF	GO:0003707∼steroid hormone receptor activity	4	0.0453508	5.003260225
GOTERM_MF	GO:0005249∼voltage-gated potassium channel activity	4	0.0494637	4.830734011

**Table 5:** Analysis of the KEGG pathway of the 281 target genes (*P* < 0.05).

Term	Count	*P* value	Fold enrichment	Bonferroni correction	Benjamini correction	*P*
hsa04151:PI3K-Akt signaling pathway	15	3.65*E* − 04	3.004348	0.058823	0.058823	0.442772
hsa04015:Rap1 signaling pathway	10	0.00302	3.290476	0.394725	0.222006	3.608333
hsa05146:Amoebiasis	7	0.003999	4.563208	0.485801	0.198856	4.752072
hsa04014:Ras signaling pathway	10	0.004906	3.057522	0.558	0.184629	5.801109
hsa04510:Focal adhesion	9	0.009149	3.018932	0.782528	0.262978	10.56663
hsa05200:Pathways in cancer	13	0.010028	2.285751	0.812318	0.243334	11.52584
hsa04915:Estrogen signaling pathway	6	0.01351	4.187879	0.895431	0.275706	15.23378
hsa04514:Cell adhesion molecules (CAMs)	7	0.015913	3.406338	0.930252	0.283128	17.70954
hsa04921:Oxytocin signaling pathway	7	0.02547	3.061392	0.986195	0.37865	26.91053
hsa04670:Leukocyte transendothelial migration	6	0.026761	3.513559	0.988922	0.362551	28.07849
hsa04512:ECM-receptor interaction	5	0.035749	3.971264	0.997625	0.42268	35.74616
hsa04974:Protein digestion and absorption	5	0.037053	3.926136	0.998103	0.406844	36.794
hsa04611:Platelet activation	6	0.038344	3.189231	0.998482	0.393022	37.81657
hsa05032:Morphine addiction	5	0.041128	3.796703	0.999062	0.392237	39.9686
hsa04530:Tight junction	6	0.046329	3.026277	0.99962	0.408424	43.80775

## Data Availability

The data used to support the findings of this study are included within the article.

## Supplementary Materials

Supplementary Materials Supplementary Table 1: predictive values of related clinical parameters. The clinicopathological parameters from patients with colon cancer were obtained from the TCGA data portal (https://portal.gdc.cancer.gov/). These data were matched with miRNA expression values, and parameters of 431 colon cancer patients were obtained. The clinicopathological parameters include gender, age, disease stage, T stage, N stage, M stage, lymphatic invasion, venous invasion, perineural invasion, treatment outcome, neoplasm recurrence, residual tumor, BMI, radiation therapy, and tumor status. Meanwhile, the prognostic value of a diversity of clinicopathological parameters was also explored. It was demonstrated by univariate Cox regression analysis that gender, disease stage, N stage, M stage, lymphatic invasion, venous invasion, treatment outcome, neoplasm recurrence, residual tumor, and tumor status were associated with the prognosis of colon cancer.
