# Supplementary material for: Study on miRNAs in Pan-Cancer of the Digestive Tract Based on the Illumina HiSeq System Data Sequencing
Source: Biomed Res Int. 2019 Oct 15;2019:8016120. doi: 10.1155/2019/8016120 (PMC6817930; doi:10.1155/2019/8016120)
Supplement: Supplementary Materials — Supplementary Table 1: predictive values of related clinical parameters. The clinicopathological parameters from patients with colon cancer were obtained from the TCGA data portal (https://portal.gdc.cancer.gov/). These data were matched with miRNA expression values, and parameters of 431 colon cancer patients were obtained. The clinicopathological parameters include gender, age, disease stage, T stage, N stage, M stage, lymphatic invasion, venous invasion, perineural invasion, treatment outcome, neoplasm recurrence, residual tumor, BMI, radiation therapy, and tumor status. Meanwhile, the prognostic value of a diversity of clinicopathological parameters was also explored. It was demonstrated by univariate Cox regression analysis that gender, disease stage, N stage, M stage, lymphatic invasion, venous invasion, treatment outcome, neoplasm recurrence, residual tumor, and tumor status were associated with the prognosis of colon cancer. [file 8016120.f1.docx]

**Supplementary table 1** The predictive values of related clinical parameters

| **Variables** |  | **Patient** | **Univariate cox analysis** | |
| --- | --- | --- | --- | --- |
|  |  | **n =431** |  |  |
|  |  |  | **HR (95% CI)** | ***P*** |
| Gender | Female | 224 | 1(reference) |  |
|  | Male | 207 | 0.902(0.605-1.347) | 0.615 |
| **Age** | <= 65 years | 179 | 1(reference) |  |
|  | > 65 years | 252 | 1.796(1.152-2.801) | **0.010** |
| **Disease stage** | I | 72 | 1(reference) |  |
|  | II | 176 | 1.890(0.729-4.900) | 0.190 |
|  | III | 121 | 3.155(1.222-8.143) | **0.018** |
|  | IV | 61 | 8.692(3.361-22.483) | **< 0.001** |
| T stage | T1 | 10 | 1(reference) |  |
|  | T2 | 72 | 0.454(0.088-2.351) | 0.347 |
|  | T3 | 293 | 1.217(0.298-4.973) | 0.784 |
|  | T4 | 55 | 3.482(0.809-14.991) | 0.094 |
| **N stage** | N0 | 253 | 1(reference) |  |
|  | N1 | 101 | 1.630(0.978-2.719) | 0.061 |
|  | N2 | 77 | 4.200(2.643-6.672) | **< 0.001** |
| **M stage** | M0 | 314 | 1(reference) |  |
|  | M1 | 61 | 4.361(2.748-6.923) | **< 0.001** |
| **Lymphatic invasion** | NO | 240 | 1(reference) |  |
|  | YES | 151 | 2.340(1.522-3.597) | **< 0.001** |
| **Venous invasion** | NO | 281 | 1(reference) |  |
|  | YES | 94 | 2.610(1.680-4.055) | **< 0.001** |
| Perineural_invasion | NO | 132 | 1(reference) |  |
|  | YES | 45 | 1.807(0.899-3.633) | 0.097 |
| **Treatment outcome** | CR+PR | 182 | 1(reference) |  |
|  | SD+PD | 41 | 14.464(6.922-30.222) | **< 0.001** |
| **Neoplasm recurrence** | NO | 282 | 1(reference) |  |
|  | YES | 78 | 2.685(1.718-4.196) | **< 0.001** |
| **Residual tumor** | R0 | 307 | 1(reference) |  |
|  | R1+R2 | 27 | 4.028(2.165-7.493) | **< 0.001** |
| BMI | NO FAT | 142 | 1(reference) |  |
|  | FAT | 89 | 0.571(0.290-1.124) | 0.105 |
| Radiation_therapy | NO | 342 | 1(reference) |  |
|  | YES | 9 | 0.631(0.087-4.551) | 0.648 |
| **Tumor status** | tumor free | 269 | 1(reference) |  |
|  | with tumor | 134 | 8.382(5.094-13.795) | **< 0.001** |
| **miR-328** | Low | 197 | 1(reference) |  |
|  | High | 234 | 1.657(1.093-2.513) | **0.017** |
